# Microwave-assisted facile synthesis of poly(luminol-*co*-phenylenediamine) copolymers and their potential application in biomedical imaging†

**DOI:** 10.1039/c8ra08373h

**Published:** 2018-11-06

**Authors:** Ufana Riaz, Sapana Jadoun, Prabhat Kumar, Raj Kumar, Nitin Yadav

**Affiliations:** Materials Research Laboratory, Department of Chemistry, Jamia Millia Islamia New Delhi-110025 India ufana2002@yahoo.co.in; Advanced Instrumentation Research Facility, Jawaharlal Nehru University New Delhi-110067 India; Cancer and Radiation Therapeutics Laboratory, School of Life Sciences, Jawaharlal Nehru University New Delhi-110067 India; Department of Chemistry, Indian Institute of Technology Delhi-110016 India

## Abstract

Conjugated copolymers have attracted much attention because of their outstanding photo-physical properties. The present work reports for the first time, microwave-assisted copolymerization of *o*-phenylenediamine with luminol using different weight ratios of the two monomers. The composition of the copolymers was confirmed by Fourier transform infrared spectroscopy (FTIR) and nuclear magnetic resonance spectroscopy (^1^H-NMR) while monomer reactivity ratios were determined using the Fineman–Ross method. Ultraviolet-visible spectroscopy revealed the variation in polaronic states upon copolymerization while X-ray diffraction (XRD) and transmission electron microscopy (TEM) analyses showed the morphology of the copolymers to be intermediate between that of the homopolymers. Confocal analysis and fluorescence studies revealed that the copolymers showed composition based blue as well as red emission which could be utilized for *in vivo* imaging of cancer cells.

## Abstract 2

Development of bioimaging agents based on poly(*o*-phenylendiamine and luminol).

## Graphical abstract

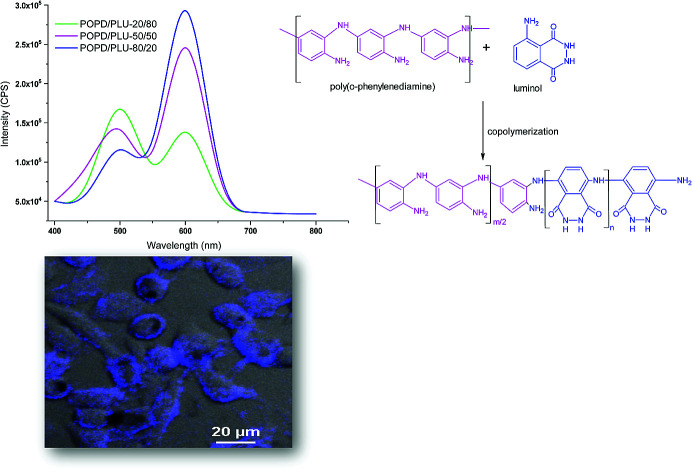

## Introduction

Stimuli responsive fluorescent nanomaterials such as quantum dots, (QDs),^1,2^ carbon nano-dots (CDs),^3,4^ polymer dots^5,6^*etc.* have been extensively investigated for their biological applications particularly in the detection and diagnosis of tumours at early stages of occurrence. QDs are well-known for their remarkable optoelectronic properties, size-tunability, high quantum yield (QY), outstanding resistance to photo-bleaching, and exceptionally large Stokes shift.^7,8^ However, most of the QDs that are effectively used as contrast imaging agents are toxic inorganic nanoparticles, which raises serious concerns regarding their potential long-term cytotoxicity under *in vivo* conditions.^9–12^ Hence, the quest for alternative biocompatible as well as non-cytotoxic materials has gained momentum.^13,14^ Several near infrared (NIR) light-emitting polymers such as polyfluorenes (PFs),^15^ polyethylene-dioxythiophene (PEDOT),^16^ polypyrrole (PPy)^17^*etc.* have been demonstrated to exhibit good biocompatibility in a number of *in vitro* and *in vivo* studies. However, efforts are still underway to develop effective conjugated polymer based theranostic agents for controllable cancer detection and diagnosis. Applications of aggregation-induced emission (AIE) of luminogens such as cyano-substituted diarylethene derivatives,^18,19^ tetraphenylethene (TPE) derivatives,^20,21^ distyrylanthracene derivatives,^22,23^ α-cyanostilbene derivatives^24,25^ in chemosensors and bio-imaging are well-documented.^26^ Polymerization-induced self-assembly (PISA) has also been investigated to prepare positive contrast agents for magnetic resonance imaging (MRI).^27,28^

Near infrared emission (NIR) provides high sensitivity towards *in vivo* measurements through minimum interference of auto-fluorescence from biological tissues and hence is regarded as a desirable technology for the diagnostic bioimaging. Hence, aith the view to develop a tunable bioimaging agent, we have for the first time, attempted to copolymerize luminol with POPD *via* microwave-assisted chemical polymerization method. Among the several polymerization techniques known, microwave-assisted synthesis offers several advantages such as solid phase synthesis, increased reaction rates, and improved product yields.^29,30^

Luminol was chosen as a co-monomer because it is commonly used as a chemiluminescence reagent in forensic medicine for selective detection of blood stains, but no work has been reported on the chemical copolymerization of luminol.^31–34^*O*-phenylenediamine (OPD) on the other hand, is an extensively explored polyaniline (PANI) derivative which exhibits outstanding photo-physical properties and fairly good water solubility.^35,36^ Copolymerization of the two monomers was carried out with a view to tailor the opto-electronic and electrochemical properties of copolymers by varying the co-monomer ratio.^37–39^

The copolymers were analysed for their spectral and morphological characteristics by employing infrared (IR), ^1^H-NMR, ultraviolet-visible (UV-Vis) spectroscopies, X-ray diffraction (XRD) and transmission electron microscopic (TEM) techniques. Fluorescence spectroscopy and confocal imaging were also carried out to explore the emission characteristics of the copolymers. Spectral studies confirmed the formation of a random copolymer exhibiting composition dependent optical characteristics. Cell viability was investigated using the methyl tetrazolium (MTT) assay and human cervical tumour (HeLa) cells. The imaging capability of the copolymers was analysed *via* live cell imaging studies.

## Experimental

Luminol, *o*-phenylenediamine, dimethyl sulfoxide and 3(4,5-dimethyl thiozol-2-yl)-2,5-diphenyl tetrazolium bromide) (MTT) assay were purchased from Sigma Aldrich, USA and were used without further purification. RPMI-1640, FBS, trypsin and antibodies were purchased from GIBCO Grand Island, New York, USA.

### Microwave-assisted synthesis of homopolymers of *o*-phenylenediamine and luminol

*O*-phenylenediamine (OPD) (1 g, 9 × 10^−2^ mol) was dispersed in deionized water (50 ml) in an Erlenmeyer flask (100 ml) containing benzoyl peroxide (BPO) (4.47993 g, 2.5 × 10^−1^ mol). The reaction mixture was exposed to microwave irradiation for 15 min at 25 °C in microwave oven (model LBP125-230, Ladd Research (USA)) as per synthesis conditions reported in our previous studies.^39^ The obtained product was then kept overnight in a deep freezer at −10 °C and was centrifuged after 24 h with distilled water to remove the unreacted impurities. The synthesized polymer was dried at 70 °C for 72 h in vacuum oven to ensure complete removal of moisture, unreacted monomer and other volatile impurities. The polymerization of luminol (PLU) was carried out in a similar manner using luminol (1 g, 5 × 10^−2^ mol) and BPO (4.47993 g, 2.5 × 10^−1^ mol). The yields obtained for POPD and PLU were 67.75% and 84.33% respectively.

### Synthesis of copolymers of *o*-phenylenediamine and luminol

To prepare copolymers of POPD : PLU, monomer (OPD) (0.5 g, 5 × 10^−2^ mol) and luminol (LUM) (0.2 g, 1 × 10^−2^ mol) were added to Erlenmeyer flask (100 ml) containing BPO (4.47993 g, 2.5 × 10^−1^ mol) homogeneously dispersed in distilled water (50 ml). The reaction mixture was exposed to microwave irradiation for 15 min at 25^°^C in microwave oven (model LBP125-230, Ladd Research (USA)) as per synthesis conditions reported in our previous studies.^39^ The synthesized copolymer centrifuged with distilled water and removal of iron was ensured by testing the filtrate with potassium ferrocyanide.^39^ The obtained copolymer was dried in vacuum oven at 70 °C (for 72 h) to ensure complete removal of water and other volatile impurities. The copolymer was designated as POPD/PLU-80/20. Similarly, the synthesis of other POPD : PLU copolymers were carried out by varying the co-monomer ratios OPD : LUM as 0.3 g, 2 × 10^−2^ mol: 0.5 g, 3 × 10^−2^ mol and 0.25 g, 2 × 10^−2^ mol: 1.63 g, 1 × 10^−2^ mol respectively. The synthesized copolymers were designated as POPD/PLU-50/50 and POPD/PLU-20/80. The percent yield obtained for the copolymers was POPD/PLU-80/20: 75.03%; POPD/PLU-50/50: 78.27% and POPD/PLU-20/80: 79.81%.

## Characterization

The viscosity average molar mass was investigated by viscosity method as reported in our previous studies.^39^ FT-IR spectra were recorded on FT-IR spectrophotometer model Shimadzu IRA Affinity-1 and the integrated absorption coefficient (∫adν̄) was determined as per reported method.^37^ UV-visible spectra were taken on UV-visible spectrophotometer model Shimadzu UV-1800 in DMSO and the molar extinction coefficient (*ε*_M_) as well as oscillator strength was calculated as per reported method.^37^^1^H-NMR spectra of the polymer solutions (prepared in CDCl_3_ 10 mg ml^−1^) were recorded on Bruker AC300. X-ray diffraction patterns were obtained using Philips PW 3710 powder diffractometer. Transmission electron micrographs (TEM) were recorded on Morgagni 268-D TEM, FEI, USA. Fluorescence spectra were recorded on fluorescence spectrophotometer model Horiba Fluorolog@3-11 in the solution state using *N*-methyl 2-pyrrolidinone (NMP) as solvent. The quantum yield was determined as per reported method.^37^ Confocal micrographs were taken on a Laser Confocal Microscope Olympus FluoView™ (*λ*_exc_ = 410 nm).

### Cell culture and MTT assay studies

The cancer cell line used for cell culture, Human cervical cancer cell (HeLa) was purchase from National Centre for Cell Science, Department of Biotechnology, Pune, India. The studies pertaining to cell proliferation, viability and imaging were carried out as per reported protocols.^31,40^

## Results and discussion

### Determination of solubility, intrinsic viscosity and viscosity average molecular weight

The solubility of PLU was observed to be higher in most of the organic solvents as compared to that of POPD. With increasing PLU content, the solubility of the copolymers was found to increase. The solubility of the copolymer POPD/PLU-20/80 was found to be highest among all the copolymers due to the presence of high luminol content, (ESI Table S1†). The intrinsic viscosity and viscosity average molecular weight 
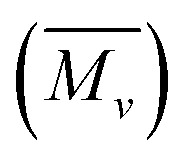
 was determined using Mark–Houwink equation.^39^ Intrinsic viscosities were observed to be 0.90 for POPD and 0.36 for PLU while for the copolymers they were calculated to be 0.73, 0.66, 0.44 for POPD/PLU-80/20, POPD/PLU-50/50, POPD/PLU-20/80 respectively (ESI Table S2†). The values of intrinsic viscosities of copolymers varied between the homopolymers. Among the copolymers, the highest 
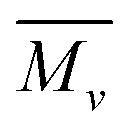
 was found to be 12 514 for the copolymer POPD/PLU-80/20 due to higher number of POPD units. Enhancement in the intrinsic viscosity was observed to be directly related to increment of molar mass but inversionally proportional to the polymerization yield (ESI Fig. S1†).

### Confirmation of copolymerization by FTIR and ^1^H-NMR studies

The ^1^H-NMR spectra of POPD, PLU and their copolymers are given in ESI, Fig. S2.† The NH protons of POPD were observed at *δ* = 6.9–7.9 ppm while the NH protons of PLU were noticed between *δ* = 7.4–8.5 ppm. The integrated areas of the NH protons of POPD (taken as 1/3) and PLU (taken as 1/4) were used for calculating their molar ratios in the copolymer. The calculated values were observed to be matching with the monomer feed ratios and therefore confirmed the copolymer composition (ESI, Table S3†).

**Scheme 1:**
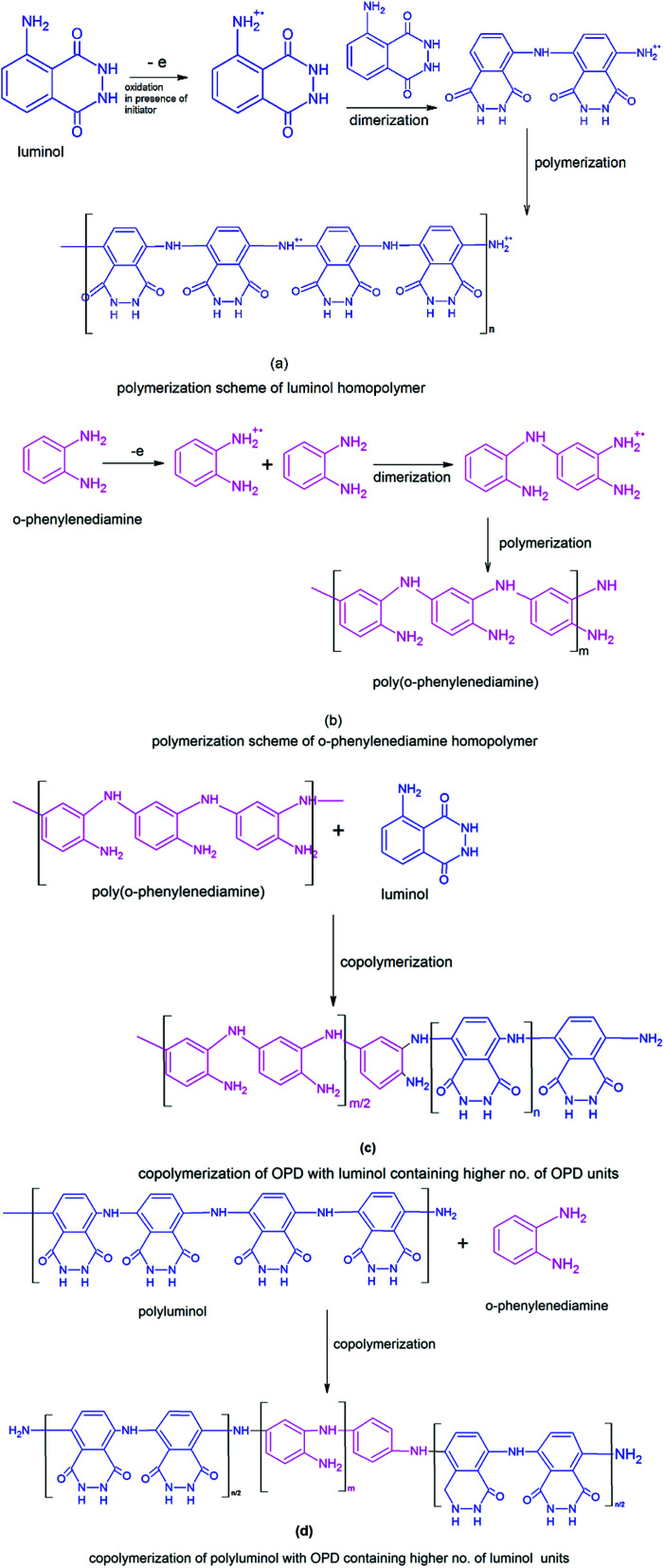
Synthesis of homopolymers and copolymers of POPD and PLU.

The monomer reactivity ratios calculated from Fineman–Ross parameters confirmed random copolymerization as shown in Scheme 1(a–d).^37^

**Fig. 1:**
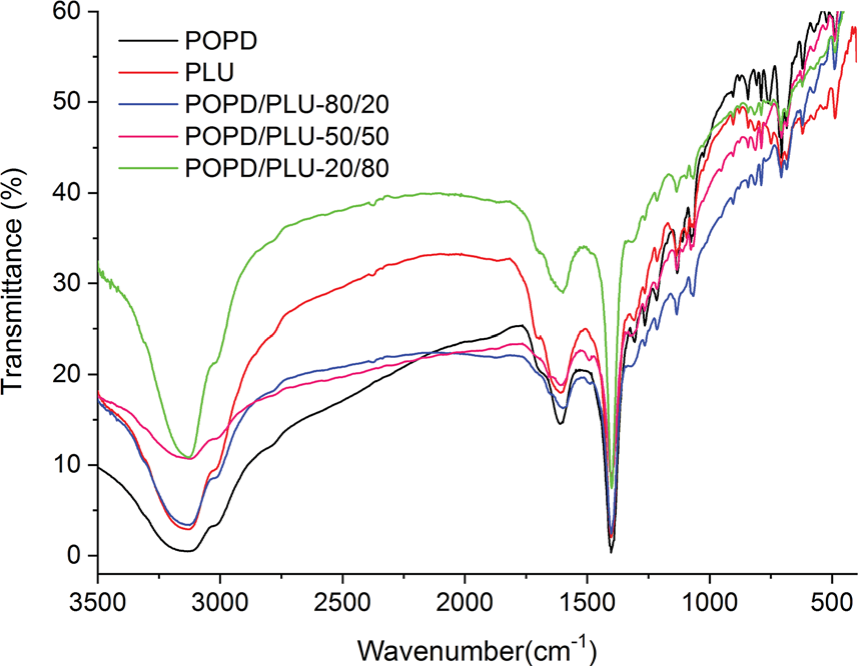
FTIR spectra of homo-polymers and copolymers of POPD/PLU.

The IR spectrum of the homopolymer POPD, Fig. 1, showed N–H stretching vibration peak at 3134 cm^−1^ associated with the presence of secondary amine (–NH–).^37^ The peak noticed at 1610 cm^−1^ was correlated to imine stretching vibration while the peaks at 1402 cm^−1^ and 1351 cm^−1^ were designated to ring puckering of the quinonoid and benzenoid units respectively.

The peak observed at 1250 cm^−1^ was assigned to CN stretching vibration. The peaks noticed at 850 cm^−1^ and 720 cm^−1^ were associated with p-substituted benzene and C–H out-of plane bending vibrations of the phenazene skeleton respectively, which confirmed the polymerization of POPD.^37,39^ The benzenoid : quinonoid (B : Q) ratio was calculated to be 3.46 indicating greater number of quinonoid units. The homopolymer PLU exhibited NH stretching vibration peak at 3132 cm^−1^ while the peak at 1668 cm^−1^ was correlated to imine stretching vibration. The peaks associated with quinonoid and benzenoid rings puckering were observed at 1402 cm^−1^ and 1350 cm^−1^ respectively and the CN stretching vibration peak was noticed at 1230 cm^−1^. The B : Q ratio was found to be 2.94 in this case indicating greater number of quinonoid units. The IR spectrum of PLU was observed to be similar to that of POPD due to the structural similarities of the two polymers. The IR spectrum of POPD/PLU-80/20, Fig. 1, showed NH stretching vibration peak at 3154 cm^−1^, while the imine stretching peak appeared at 1668 cm^−1^. The quinonoid and benzenoid peaks were observed at 1598 cm^−1^ and 1402 cm^−1^ respectively and the B : Q ratio was calculated to be 2.41. The value was noticed to be intermediate of the homopolymers. The NH stretching vibration peak for POPD/PLU-50/50, Fig. 1, was observed at 3124 cm^−1^ revealing a shift of about 10 cm^−1^ as compared to that of pristine POPD whereas the imine stretching peak was noticed at 1633 cm^−1^. The B : Q ratio in this case was 1.65 which was lower than that of POPD/PLU-80/20. Similarly, POPD/PLU-20/80 showed the NH stretching vibration peak at 3134 cm^−1^ as noticed in POPD but the imine stretching peak was noticed at 1668 cm^−1^ which was comparable to the one observed in PLU. The B : Q ratio was observed to be 1.17 which was lowest among all the copolymers. The NH peak area (∫adν̄) value was found to be 1088 for POPD and 853 for PLU. The ∫adν̄ values for POPD/PLU-80/20, POPD/PLU-50/50 and POPD/PLU-20/80 were calculated to be 741, 568 and 380 respectively. The ∫adν̄ values were noticed to decrease with the increase in the PLU content. Likewise, the ∫adν̄ values for the imine stretching peak were observed to be 97 for POPD and 28 for PLU while for POPD/PLU-80/20, POPD/PLU-50/50 and POPD/PLU-20/80, they were observed to be 44, 52 and 25 respectively, showing a similar trend of decrease in the area with the increase in the PLU content as seen in case of the NH peak. It can therefore be concluded that copolymerization of POPD and PLU was observed to be composition dependent as the NH as well as the imine stretching peaks showed variation in the ∫adν̄ values which could be well correlated to the co-monomer content in the copolymer.

### Influence of copolymerization on morphology confirmed by XRD and TEM studies

**Fig. 2:**
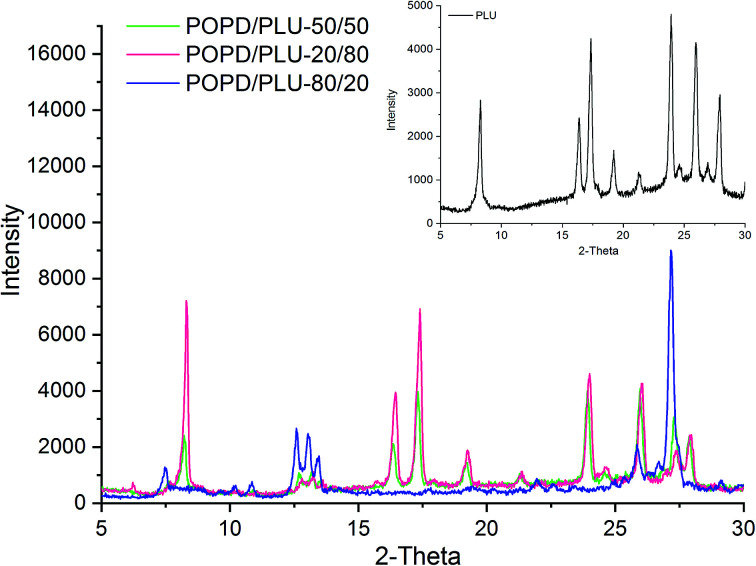
XRD of PLU and POPD/PLU copolymers.

The XRD pattern of PLU (inset), Fig. 2, exhibited prominent peaks at 2*θ* = 7.5°, 16.3°, 18.2°, 19.8°, 21.2°, 24.3°, 26.2° and 28.3° revealing a highly crystalline structure. The planes observed at (020), (010), (100), (110) and (011) revealed a pseudo orthorhombic lattice type for the polymer.^37^ The XRD pattern of POPD has been reported in our previous work which revealed peaks at 2*θ* = 8.33°, 18.5°, 19.78°, 23.5° and 28.08° with limited planes at (010), (110) and (011).^37^

**Fig. 3:**
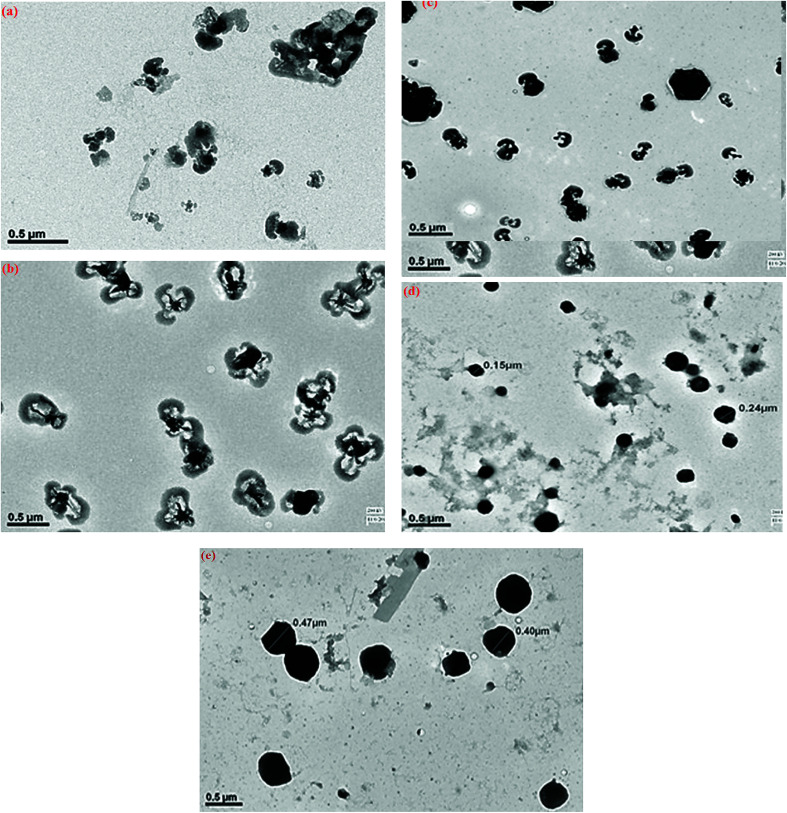
TEM micrographs of (a) POPD, (b) PLU, (c) POPD/PLU-80/20, (d) POPD/PLU-50/50and (e) POPD/PLU-20/80.

The TEM of PLU, Fig. 3(a), revealed distorted cluster formation and the average size of the particles was observed to be 200 nm. The TEM of POPD, Fig. 3(b), showed distorted sea-shell like morphology and the particles were observed to have a dense core surrounded by thin layer of film. The copolymer POPO/PLU-80/20, Fig. 3(c), revealed a mixed morphology of dense hexagonal and sea-shell like particles matching those observed in case of pristine POPD. The copolymer POPO/PLU-50/50, Fig. 3(d), showed formation of compact spherical particles, with an average diameter of 160 nm while, the TEM of POPO/PLU-20/80, Fig. 3(e), showed formation of large spherical particles exhibiting an average diameter of 470 nm. A mixed morphology was observed for the copolymers depending upon the PLU/POPD content. The copolymers revealed peaks associated with both the homopolymers along with minor variations in the 2*θ* values of some of the peaks. All the peaks were found to be well disposed indicating a highly organized and crystalline morphology of the copolymers. POPD/PLU-80/20 displayed peaks corresponding to PLU of lowest intensity while the intensity for the peaks corresponding to POPD was pronounced.

### Variation in the UV and fluorescence characteristics upon copolymerization

**Fig. 4:**
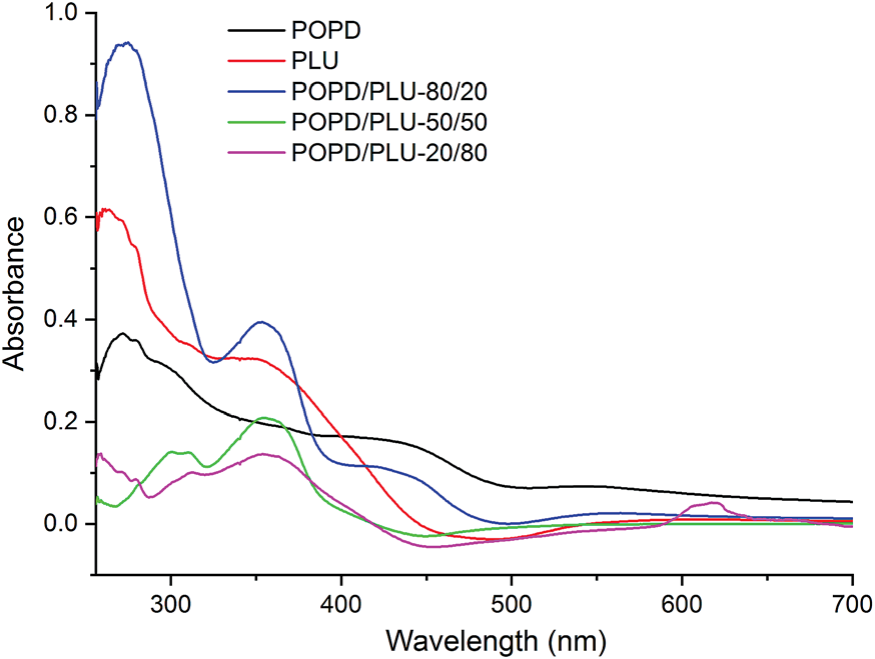
UV-visible spectra of POPD, PLU and its copolymers.

The UV-visible spectra of POPD, PLU and its copolymers are depicted in Fig. 4. The peaks observed at 280 nm and 425 nm for POPD were assigned to π–π* transition and polaronic transition respectively as reported in our previous studies.^37^ The peaks associated with similar transition were observed at 270 nm and 375 nm in PLU.^37,38^ Interestingly, the UV-visible spectra of copolymers revealed peaks corresponding to both POPD and PLU. POPD/PLU-80/20 copolymer revealed peaks at 280 nm, 375 nm and 425 nm confirming that the individual electronic states of the homopolymers were retained even after copolymerization.

The intensity of the peaks corresponding to POPD was higher as compared to that of PLU confirming higher content of the former in the copolymer. Likewise, the UV spectrum of POPD/PLU-50/50 showed peaks at 300 nm, 375 nm confirming the presence of PLU in the copolymer.^38^ The peak at 425 nm associated with POPD was observed to be missing. Similarly in case of POPD/PLU-20/80, a broad hump was noticed at 350 nm and a small peak was noticed around 610 nm. For POPD/PLU-80/20, the ∫adν̅ and the *ε*_M_ values were found to be 82.32 ± 0.03 and 1437 respectively while for copolymer POPD/PLU-50 : 50, ∫adν̅ and *ε*_M_ values were noticed to be 64.13 ± 0.01, 1077 respectively. The ∫adν̅ and *ε*_M_ values for POPD/PLU-20/80 were found to be 40.73 ± 0.02 and 754 respectively. The oscillator strength was observed to be highest for pristine POPD. The oscillator strength decreased from 0.012 ± 0.03 to 0.0060 ± 0.03 as the copolymer composition of POPD/PLU varied from 80/20 to 20/80. Thus, the progressive change in the optical properties with the copolymer composition indicated random copolymerization (Table 1).

**Table 1:** UV data of POPD, PLU and their copolymers, data represent mean ± SD, *n* = 3

Sample	*λ*_max_ (nm)	∫adν̄ integrated absorption coefficient, cm^−2^	Molar extinction coefficient (*ε*_M_) (M^−1^ cm^−1^)	Oscillator strength
POPD	425	142.46 ± 0.02	2515	0.022 ± 0.02
PLU	375	25.93 ± 0.03	323	0.004 ± 0.01
POPD/PLU-80/20	425	82.32 ± 0.03	1437	0.012 ± 0.03
POPD/PLU-50/50	375	64.13 ± 0.01	1077	0.009 ± 0.04
POPD/PLU-20/80	610	40.73 ± 0.02	754	0.006 ± 0.03

**Fig. 5:**
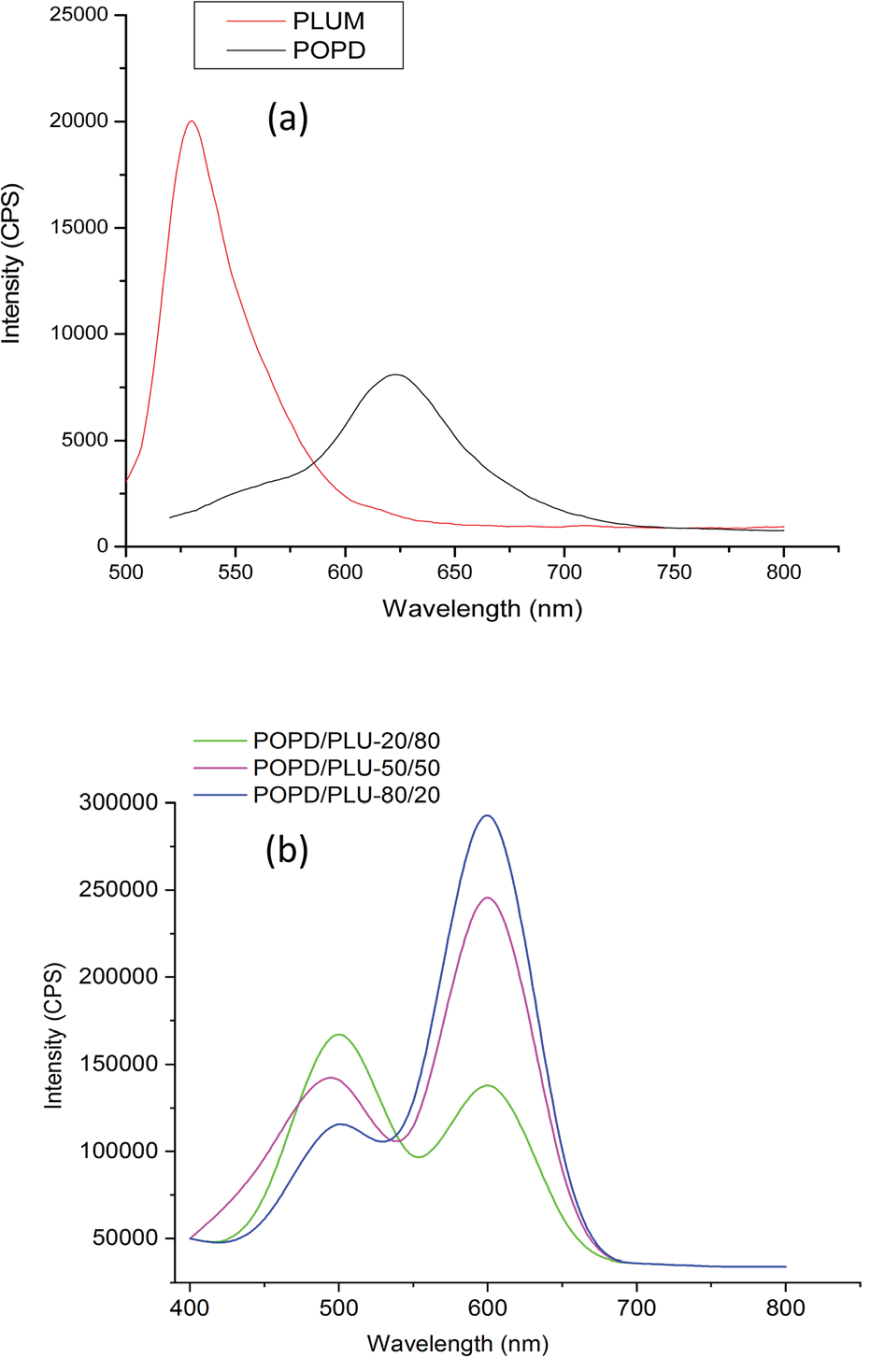
Fluorescence spectra of (a) POPD, PLU (b) POPD/PLU copolymers.

The fluorescence emission spectrum of POPD, Fig. 5(a), showed an intense broad peak centered at 620 nm upon excitation at 350 nm while the spectrum of PLU showed a pronounced peak at 525 nm at similar excitation wavelength which was attributed to S_1_ → S_0_ transition.^37,39^ The copolymers revealed broad emission peaks at 500 nm and 610 nm.

The intensity of the peak at 610 nm associated with POPD was observed to increase with the increase in the number of POPD units in the copolymer. For POPD/PLU-80/20, the emission peak at 500 nm peak associated with PLU was observed to be lower, while the peak related to POPD was noticed to have highest intensity among all the copolymers due to higher number of POPD units in this copolymer. The intensity of the 500 nm peak was observed to increase with the increase in the number of PLU units in the copolymer. The intensity of the emission peak at 500 nm was observed to be 110 000 cps while the intensity for the emission peak at 610 nm was noticed to be 280 000 cps. The copolymer POPD/PLU-50/50 revealed an emission intensity of 245 000 cps for the peak at 620 nm while the intensity of the emission peak at 500 was found to be 145 000 cps. The copolymer POPD/PLU-20/80, showed emission intensities of 160 000 cps and 125 000 cps for 500 nm and 610 nm peaks respectively. The *Φ* values were calculated by taking Rhodamine B (RhB) as reference, Table 2.

**Table 2:** Quantum yield values for homopolymers and copolymers of POPD and PLU in NMP

Sample	*λ*_Em_ (nm)	Integrated area (*I*_samp_)	Quantum yield (*Φ*)
PLU	525	3.57 × 10^8^	0.14 ± 0.05
POPD	620	2.42 × 10^9^	0.36 ± 0.03
POPD/PLU-20/80	500	1.79 × 10^8^	0.12 ± 0.04
	610	2.34 × 10^7^	0.22 ± 0.06
POPD/PLU-50/50	500	2.14 × 10^8^	0.10 ± 0.05
	610	2.18 × 10^8^	0.31 ± 0.03
POPD/PLU-80/20	500	1.34 × 10^8^	0.09 ± 0.04
	610	2.32 × 10^9^	0.34 ± 0.02

For POPD, the ∫adν̅ value of the emission peak at 620 nm was calculated to be 2.42 × 10^9^ and the *Φ* value was found to be 0.36 ± 0.03. Similarly for PLU, the ∫adν̅ for the emission peak at 525 nm was calculated to be 3.57 × 10^8^ and the *Φ* value was found to be 0.14 ± 0.05. The ∫adν̅ value for the emission peak at 500 nm in case of POPD/PLU-20/80 was found to be 1.79 × 10^8^ while for the peak at 610 nm, it was calculated to be 2.34 × 10^7^. The *Φ* values were calculated as 0.12 ± 0.04 and 0.22 ± 0.06 corresponding to the emission peaks observed at 500 nm and 610 nm respectively. The copolymers POPD/PLU-50/50 and POPD/PLU-80/20 showed ∫adν̅ values 2.14 × 10^8^, 2.18 × 10^8^, 1.34 × 10^8^ and 2.32 × 10^9^ for the peaks at 500 nm and 610 nm respectively while the *Φ* values were found to be 0.10 ± 0.05, 0.31 ± 0.03, 0.09 ± 0.04 and 0.34 ± 0.02 respectively. The variation in the fluorescence intensity matched well with the ∫adν̅ values. As the ratio of POPD in the copolymer increased, quantum yield increased from 0.22 ± 0.06 to 0.34 ± 0.02. Quantum yield of POPD as well as PLU were noticed to be proportional to their ratios in the copolymer. Hence, composition dependant fluorescence properties were attained upon varying the loading of POPD as well as PLU.

### Confocal studies

**Fig. 6:**
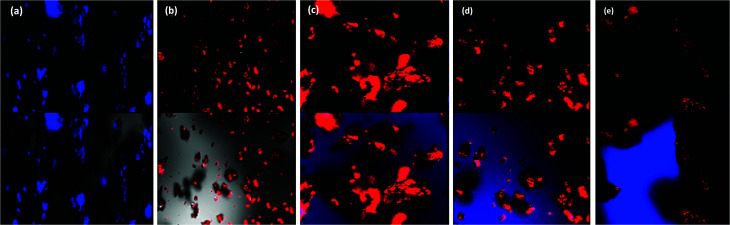
Confocal micrographs of (a) PLU, (b) POPD, (c) POPD/PLU-80/20, (d) POPD/PLU-50/50, (e) POPD/PLU-20/80.

The confocal analysis of the homopolymers and copolymers was carried out in solution state and is shown in Fig. 6. The confocal micrograph of PLU, Fig. 6(a), showed intense blue coloured particles. The fluorescence emission spectrum of PLU also revealed an emission peak around 525 nm. POPD was noticed to emit intense red coloured tiny particles as the polymer revealed fluorescence emission peak at 620 nm, Fig. 6(b).

The copolymer POPD/PLU/80/20, Fig. 6(c), revealed intense red coloured particles with a blue region of PLU particles. Similarly, POPD/PLU-50/50, Fig. 6(c) and POPD/PLU-20/80, Fig. 6(d) showed the presence of intense red nanoparticles which could be correlated to POPD while the PLU region was found to blue in colour. The morphology was found to consist of densely distributed aggregates of irregularly shaped fine particles of POPD dispersed in the PLU matrix. The regions of red and blue emission by the particles were found to vary with the composition of the copolymer which clearly confirmed that the homopolymers retained their photo = physical properties even upon copolymerization.

### Cytotoxicity and imaging of tumor cells

**Fig. 7:**
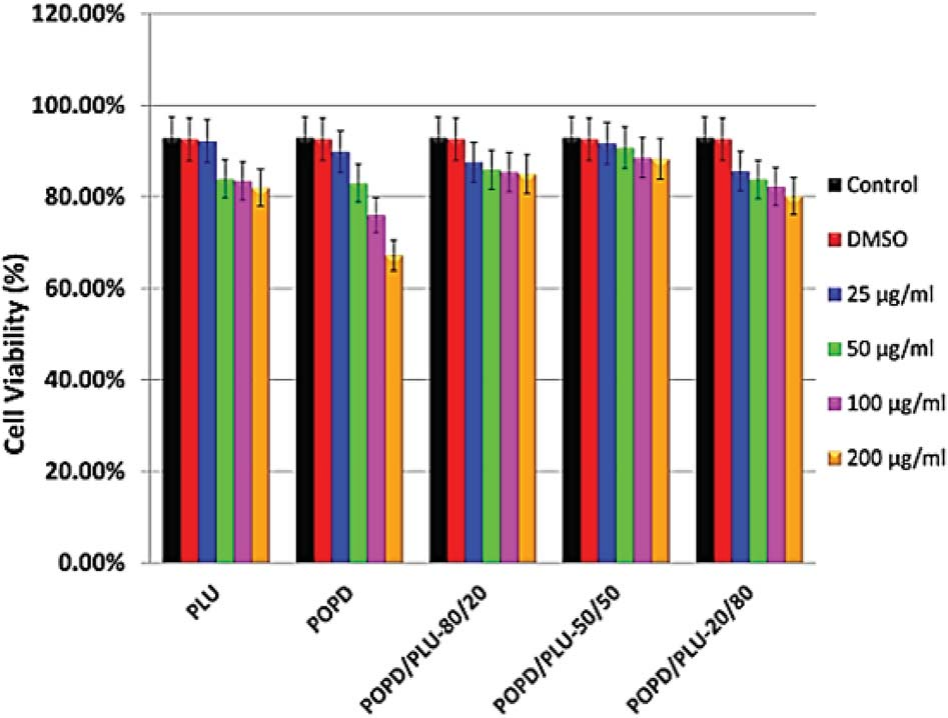
Cytotoxicity analysis of POPD, PLU and its copolymers.

HeLa cells were used to examine cellular toxicity, which included normal as well as tumor cells. The effects of homopolymers and copolymers on cell viability and proliferation were measured using the MTT assay, which is based on the reduction of the yellow tetrazolium salt MTT by metabolically active cells, resulting in purple formazan crystals. Among the homopolymers, neither PLU nor POPD revealed any toxic effect over the concentration range of 25–50 μg ml^−1^. Interestingly, the copolymers did not reveal any toxic effect up to the concentration of 200 μg ml^−1^, Fig. 7. Only in case of pure POPD, the concentration of 200 μg ml^−1^ showed to slightly block the cell viability. The cytotoxicity of the homopolymers as well as copolymers was observed to be time independent.

The copolymers were found to be non-toxic at concentrations as high as 200 μg ml^−1^ in all tested cell lines. The copolymer POPD/PLY-50/50 showed results quite comparable to the control group. The tumor cell lines clearly appeared to be able to tolerate exposure to copolymers through high metabolic activity during *in vitro* cytotoxicity tests and revealed capability of maintaining their cell proliferation and membrane integrity during exposure to higher dosages of copolymers.

**Fig. 8:**
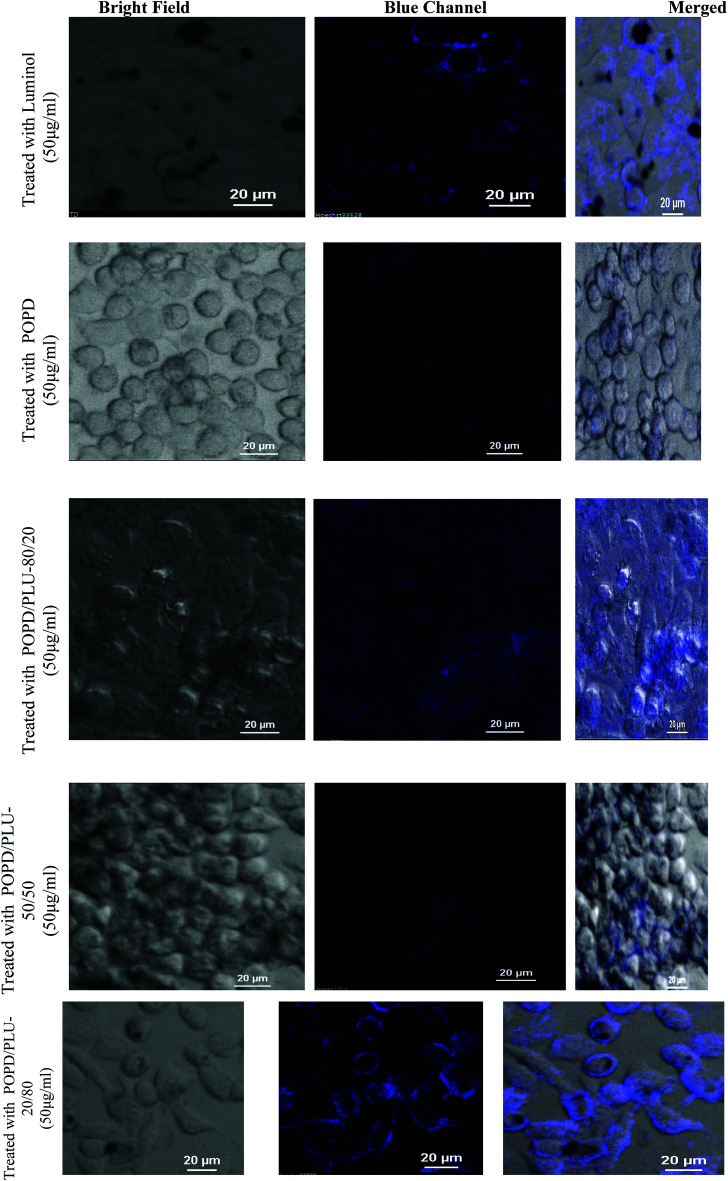
Fluorescence imaging of HeLa cells incubated with the copolymers for 4 h.

The cellular uptake of POPD, PLU and its copolymers by the HeLa cells was visualized *via* confocal microscopic imaging, Fig. 8. The studies revealed that blue luminescence was observed by the HeLa cells treated with homopolymers as well as copolymers for a period of 4 h, Fig. 8. Furthermore, the internalization of the pristine polymers and copolymers after 4 h of treatment was clearly observed in case of HeLa cells, which showed high expression levels. An intensive fluorescence signal was observed on the surface and in the cytoplasm of the HeLa cells after treatment with the PLU and POPD/PLU-20/80. In contrast, only slight fluorescence signals were observed in the HeLa cells under similar experimental conditions using POPD and copolymers containing higher POPD content. These results demonstrated the effective targeting specificity of the copolymers containing higher PLU content towards HeLa cells. To get a better insight, time-dependent confocal microscopy studies were carried out for POPD, PLU and POPD/PLU-20/80 treated HeLa cells. It was observed that the copolymer POPD/PLU-20/80 showed a better contrast even after 24 h (ESI Fig. S3†). The time dependent confocal analysis confirmed that PLU and POPD/PLU-20/80 could be used as a novel fluorescent indicator to study the absorption, transportation processes inside the mammalian cells.

## Conclusion

Microwave-assisted copolymerization of *o*-phenylenediamine with luminol was successfully carried out using different weight ratios of the two monomers. ^1^H-NMR studies confirmed random copolymerization while UV-visible and fluorescence studies showed composition dependant photo-physical properties. X-ray diffraction (XRD) and transmission electron microscopy (TEM) analyses confirmed the morphology of copolymers to be dependent on the ratio of the two monomers in the copolymer. Confocal analysis also revealed composition dependant emission of the copolymers which was found to be intense red/blue depending upon the content of POPD/PLU in the copolymer. MTT assay studies confirmed that the homopolymers as well as the copolymers were non-toxic and could be safely used for *in vivo* imaging of cancer cells. The confocal images of the homopolymers/copolymer modified HeLa cells revealed blue luminescence and the internalization of PLU and POPD/PLU-20/80 showed high expression levels which could be used as an efficient nanoprobe for imaging of various kinds of tumors.

## Conflicts of interest

On behalf of all authors, the corresponding author states that there is no conflict of interest.

## Supplementary Material

RA-008-C8RA08373H-s001
