# Supplementary material for: Microwave-assisted facile synthesis of poly(luminol-co-phenylenediamine) copolymers and their potential application in biomedical imaging
Source: RSC Adv. 2018 Nov 6;8(65):37165–75. doi: 10.1039/c8ra08373h (PMC9089407; doi:10.1039/c8ra08373h)
Supplement: RA-008-C8RA08373H-s001 [file RA-008-C8RA08373H-s001.pdf]

Electronic Supplementary Material (ESI) for RSC Advances.
This journal is © The Royal Society of Chemistry 2018

Supporting information

Microwave-assisted Facile Synthesis of Poly(lumniol-co-phenylenediamine) Copolymers and their Potential Application in Biomedical Imaging

Ufana Riaz[a]*, Sapana Jadoun[a], Prabhat Kumar[b],Raj Kumar[c] and Nitin Yadav[d]

[a]Materials Research Laboratory Department of Chemistry,Jamia Millia Islamia,New Delhi-110025, India, [b] Advanced Instrumentation Research Facility, Jawaharlal Nehru University, New Delhi- 110067, [c]School of Life Sciences, Jawaharlal Nehru University, New Delhi-110067,[d]Department of Chemistry, Indian Institue of Technology ,Delhi-110016

*corresponding author email: ufana2002@yahoo.co.in

Table S1.Solubility of homopolymers and copolymers in different solvents

Polymer/copolymer	NMP	DMSO	THF	Methanol	Acetone	Sulphuric Acid
POPD	ES	ES	PS	PS	PS	ES
PLU	ES	ES	ES	ES	PS	ES
80/20(POPD/PLU)	ES	ES	PS	PS	PS	ES
50/50(POPD/PLU)	ES	ES	PS	PS	PS	ES
20/80(POPD/PLU)	ES	ES	PS	PS	PS	ES

(ES- Easily soluble; PS- partially soluble)

Table S2. Intrinsic Viscosities and Viscosity Average molar mass of synthesized polymers

Polymer	Intrinsic Viscosity(η)	Viscosity Average Molar Mass(M_v)
POPD	0.90	14570
PLU	0.36	7498
POPD/PLU- 80/20	0.73	12514
POPD/PLU- 50/50	0.60	10795
POPD/PLU -20/80	0.44	8658

Table 3 Molar feed ratios determined by ^{1}H-NMR and Fineman Ross parameters

Molar feed ratio (OPD:LUM)	Monomer in Feed		f $=f_1/f_2$	Molar ratio as determined by ^{1}H-NMR*		F $=F_1/F_2$	Fineman Ross Parameters	
	f_1	f_2		F_1	F_2		f(F-1)/F	f^2/F
80/20 *(73/27)	0.740	0.112	6.60	0.675	0.153	4.41	5.10	9.8
50/50 *(45/55)	0.462	0.282	1.63	0.417	0.311	1.34	0.414	1.98
20/80 *(15/85)	0.185	0.452	0.40	0.138	0.480	0.28	1.02	0.57

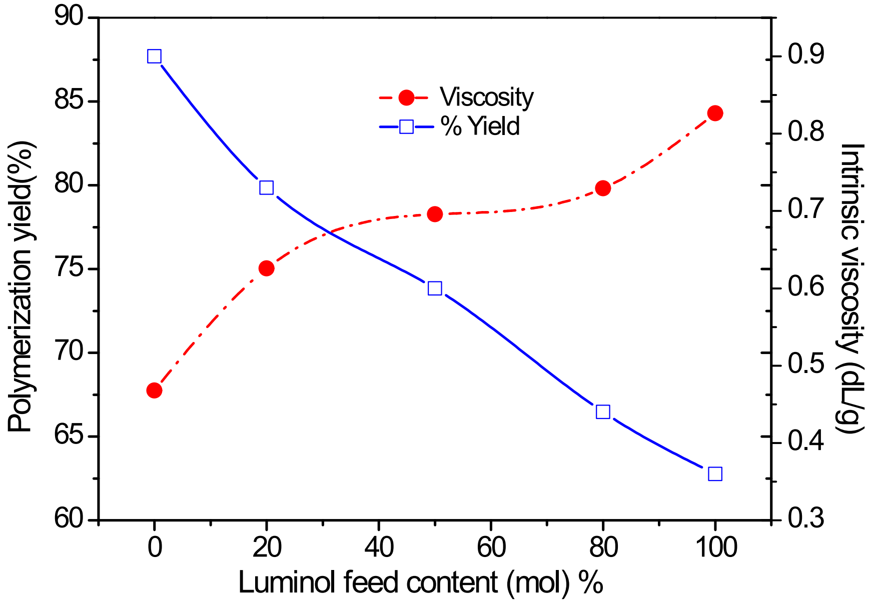

Figure S1. Influence of Luminol (LU) feed content on polymerization yield and intrinsic viscosity of OPD/LU copolymers

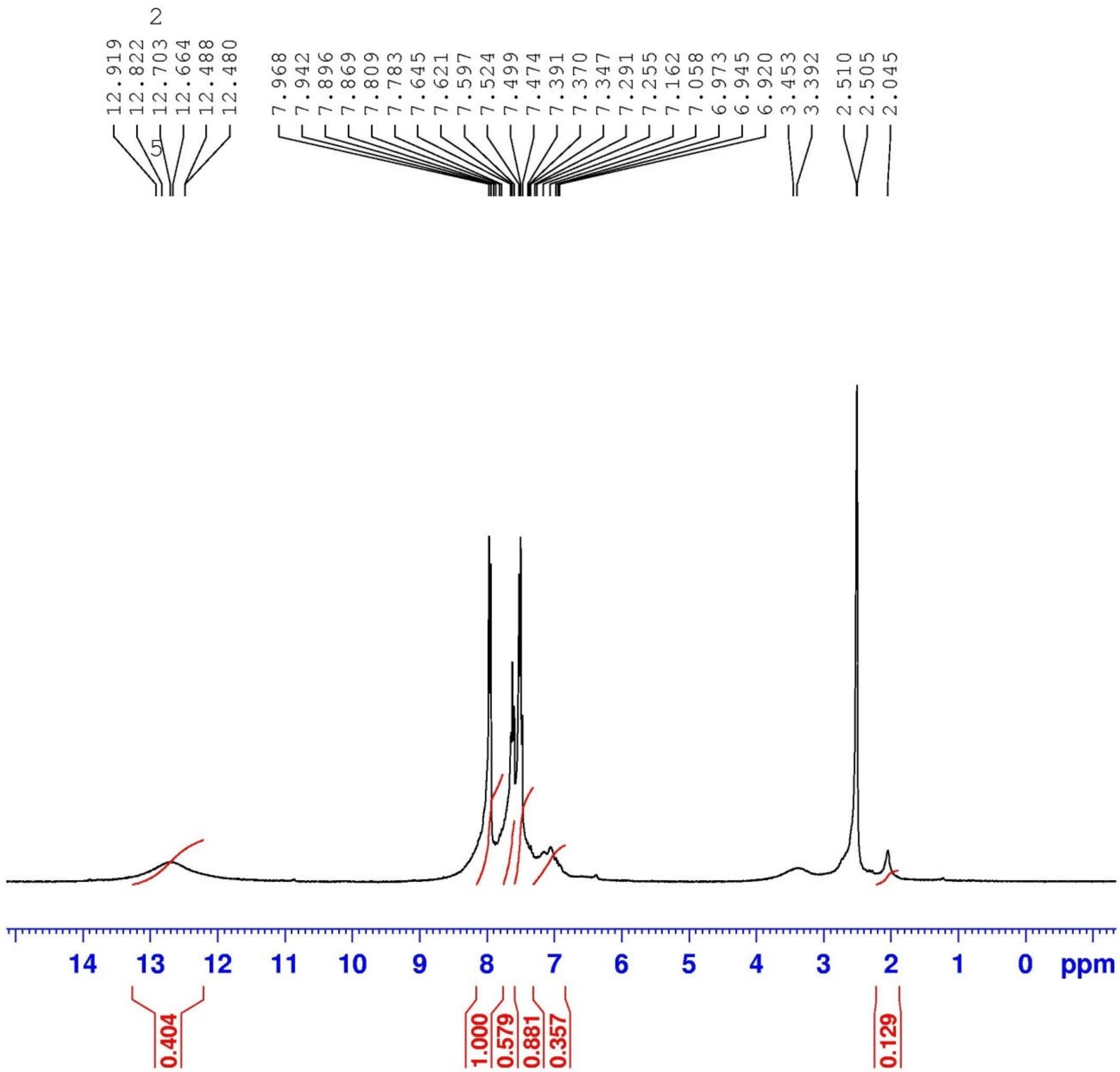
12.919
12.822
12.703
12.664
12.488
12.480
7.968
7.942
7.896
7.869
7.809
7.783
7.645
7.621
7.597
7.524
7.499
7.474
7.391
7.370
7.347
7.291
7.255
7.162
7.058
6.973
6.945
6.920
3.453
3.392
2.510
2.505
2.045
14 13 12 11 10 9 8 7 6 5 4 3 2 1 0 ppm
0.404
1.000
0.579
0.881
0.357
0.129

(a)

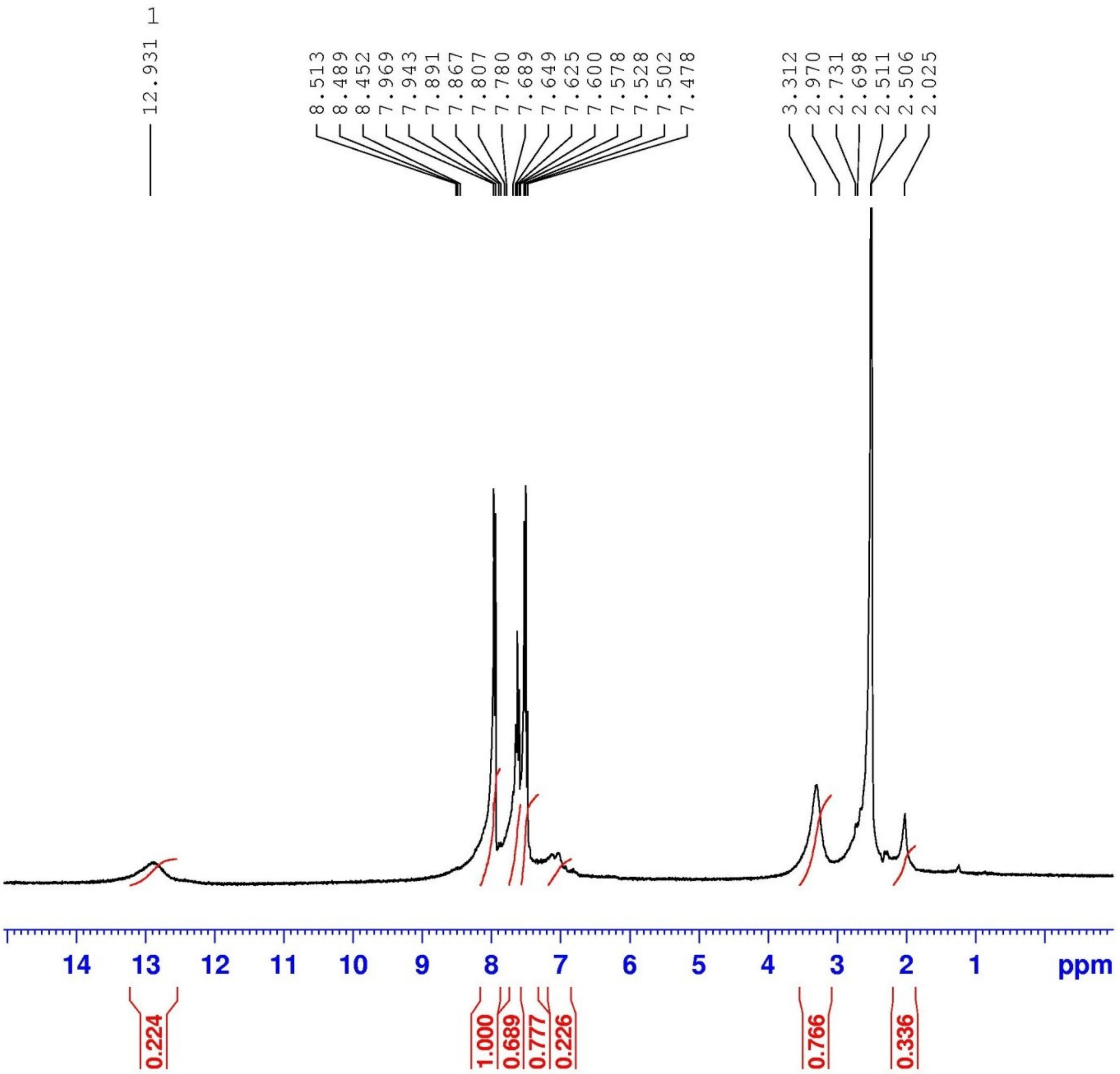

1
12.931
8.513
8.489
8.452
7.969
7.943
7.891
7.867
7.807
7.780
7.689
7.649
7.625
7.600
7.578
7.528
7.502
7.478
3.312
2.970
2.731
2.698
2.511
2.506
2.025
14
13
12
11
10
9
8
7
6
5
4
3
2
1
ppm
0.224
1.000
0.689
0.777
0.226
0.766
0.336

(b)

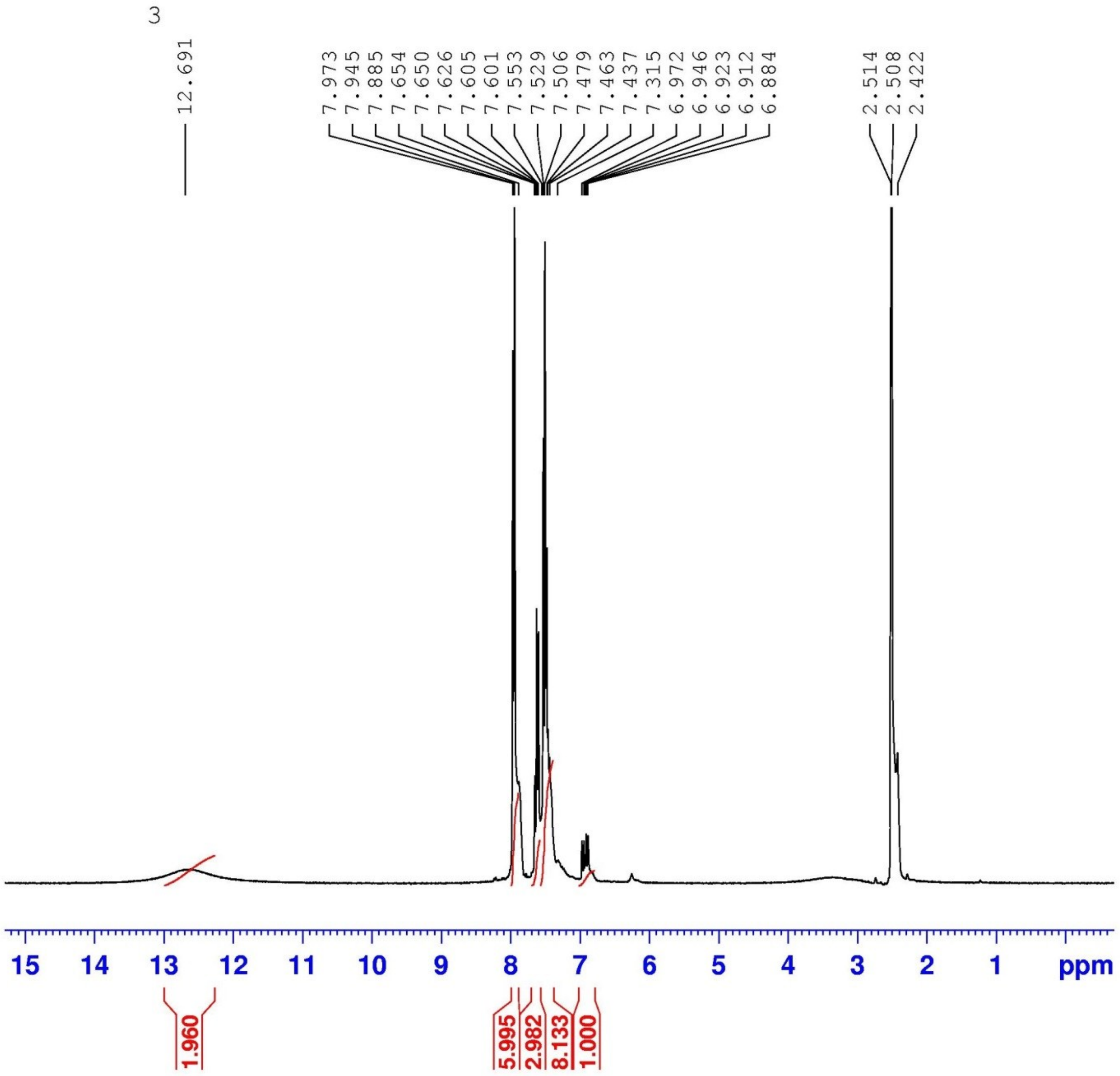
3
12.691
7.973
7.945
7.885
7.654
7.650
7.626
7.605
7.601
7.553
7.529
7.506
7.479
7.463
7.437
7.315
6.972
6.946
6.923
6.912
6.884
2.514
2.508
2.422
15
14
13
12
11
10
9
8
7
6
5
4
3
2
1
ppm
1.960
5.995
2.982
8.133
1.000

(c)

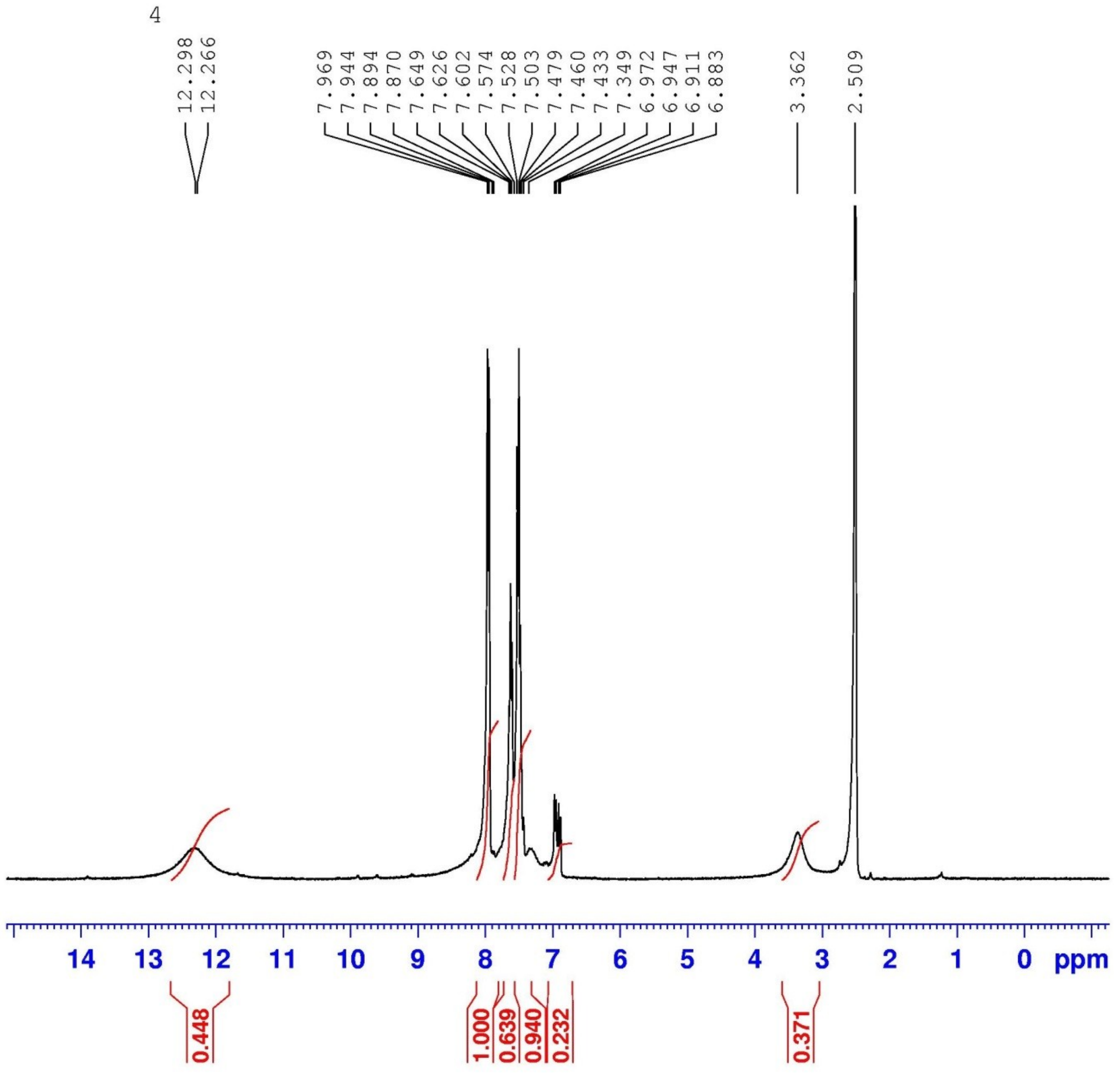
4
12.298
12.266
7.969
7.944
7.894
7.870
7.649
7.626
7.602
7.574
7.528
7.503
7.479
7.460
7.433
7.349
6.972
6.947
6.911
6.883
3.362
2.509
14 13 12 11 10 9 8 7 6 5 4 3 2 1 0 ppm
0.448
1.000
0.639
0.940
0.232
0.371

(d)

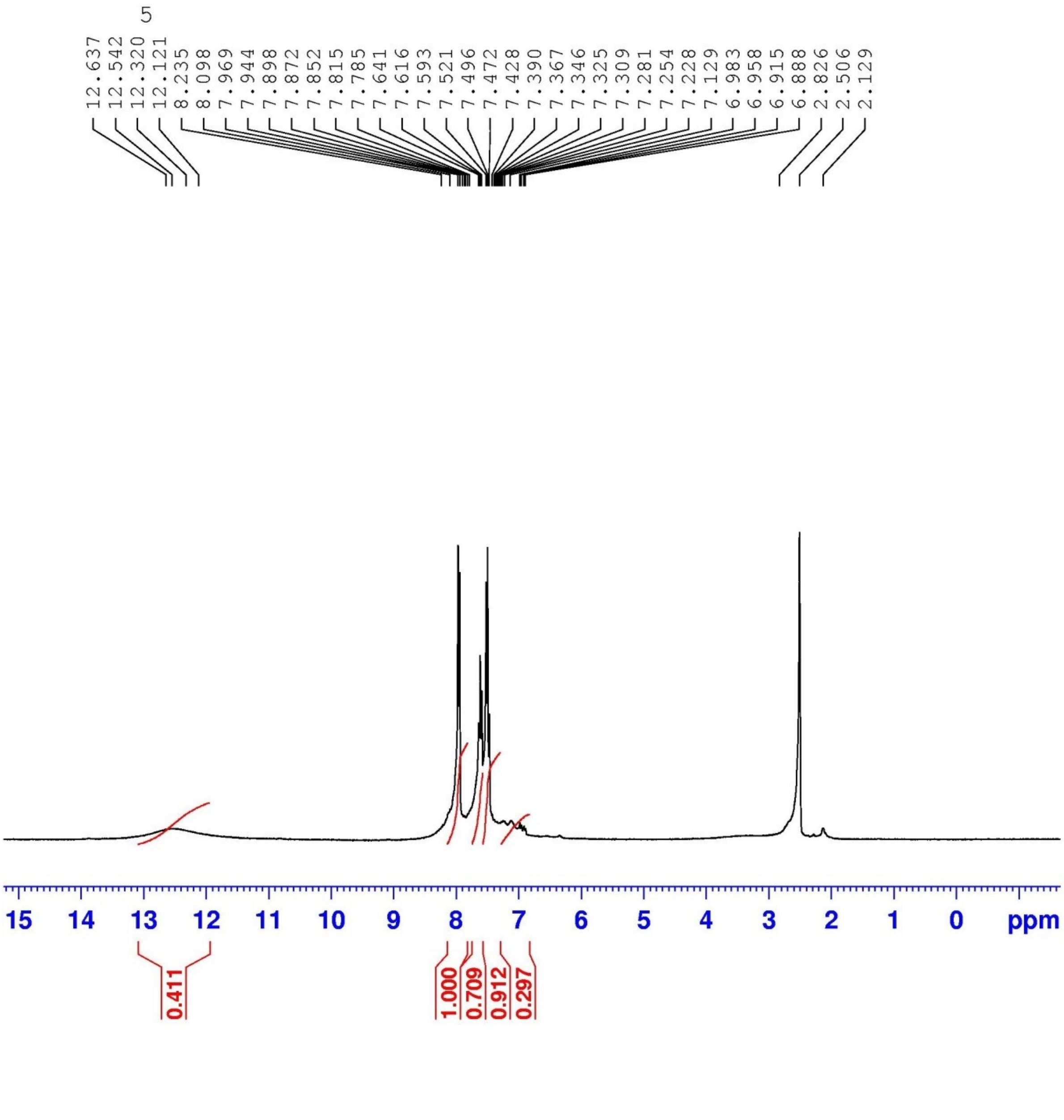

(e)

Figure S2 ^{1}H-NMR spectra of (a) POPD, (b) PLU, (c) POPD/PLU-80/20, (d) POPD/PLU-50/50, (e) POPD/PLU-20/80

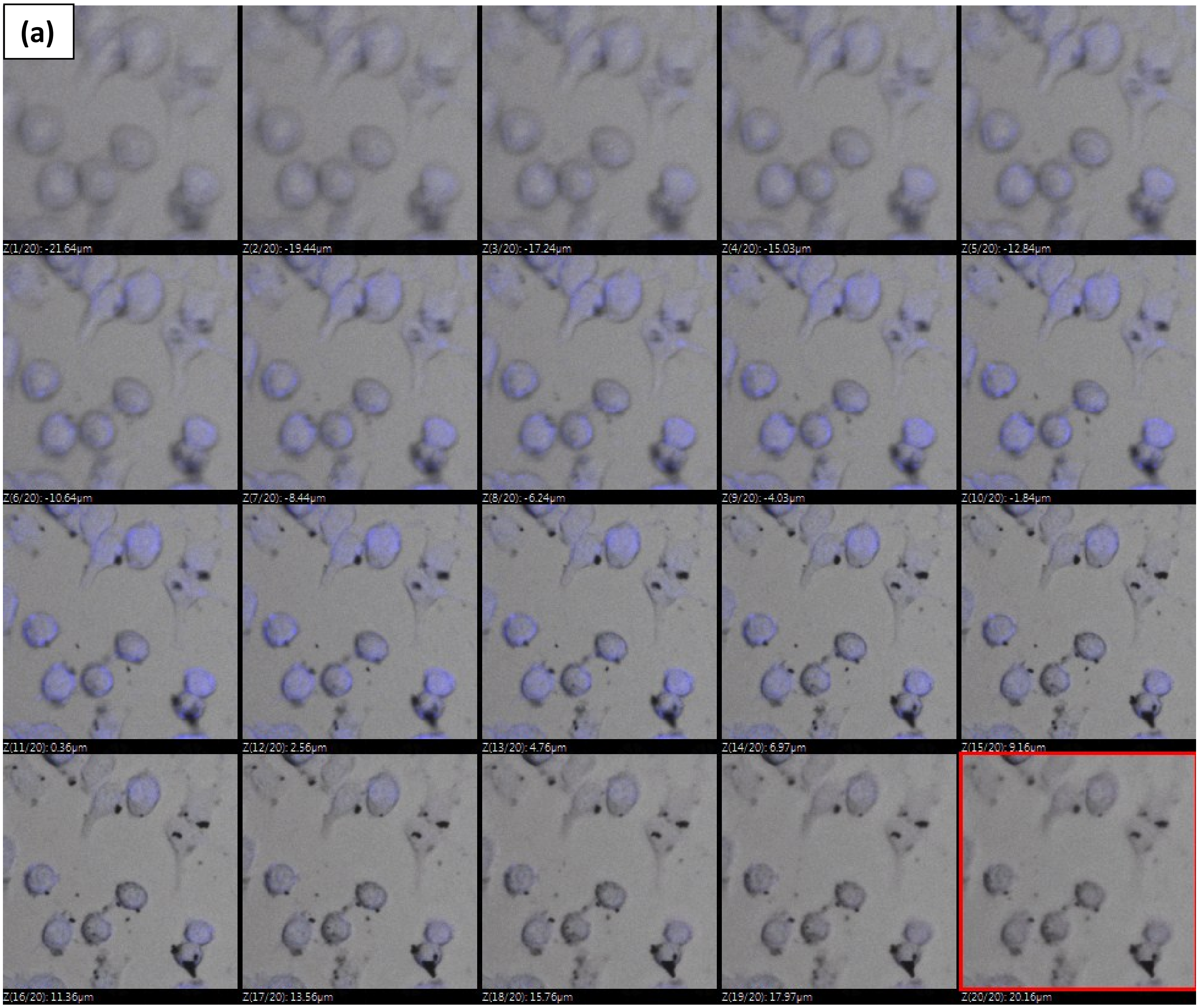
(a)
Z(1/20): -21.64µm
Z(2/20): -19.44µm
Z(3/20): -17.24µm
Z(4/20): -15.03µm
Z(5/20): -12.84µm
Z(6/20): -10.64µm
Z(7/20): -8.44µm
Z(8/20): -6.24µm
Z(9/20): -4.03µm
Z(10/20): -1.84µm
Z(11/20): 0.36µm
Z(12/20): 2.56µm
Z(13/20): 4.76µm
Z(14/20): 6.97µm
Z(15/20): 9.16µm
Z(16/20): 11.36µm
Z(17/20): 13.56µm
Z(18/20): 15.76µm
Z(19/20): 17.97µm
Z(20/20): 20.16µm

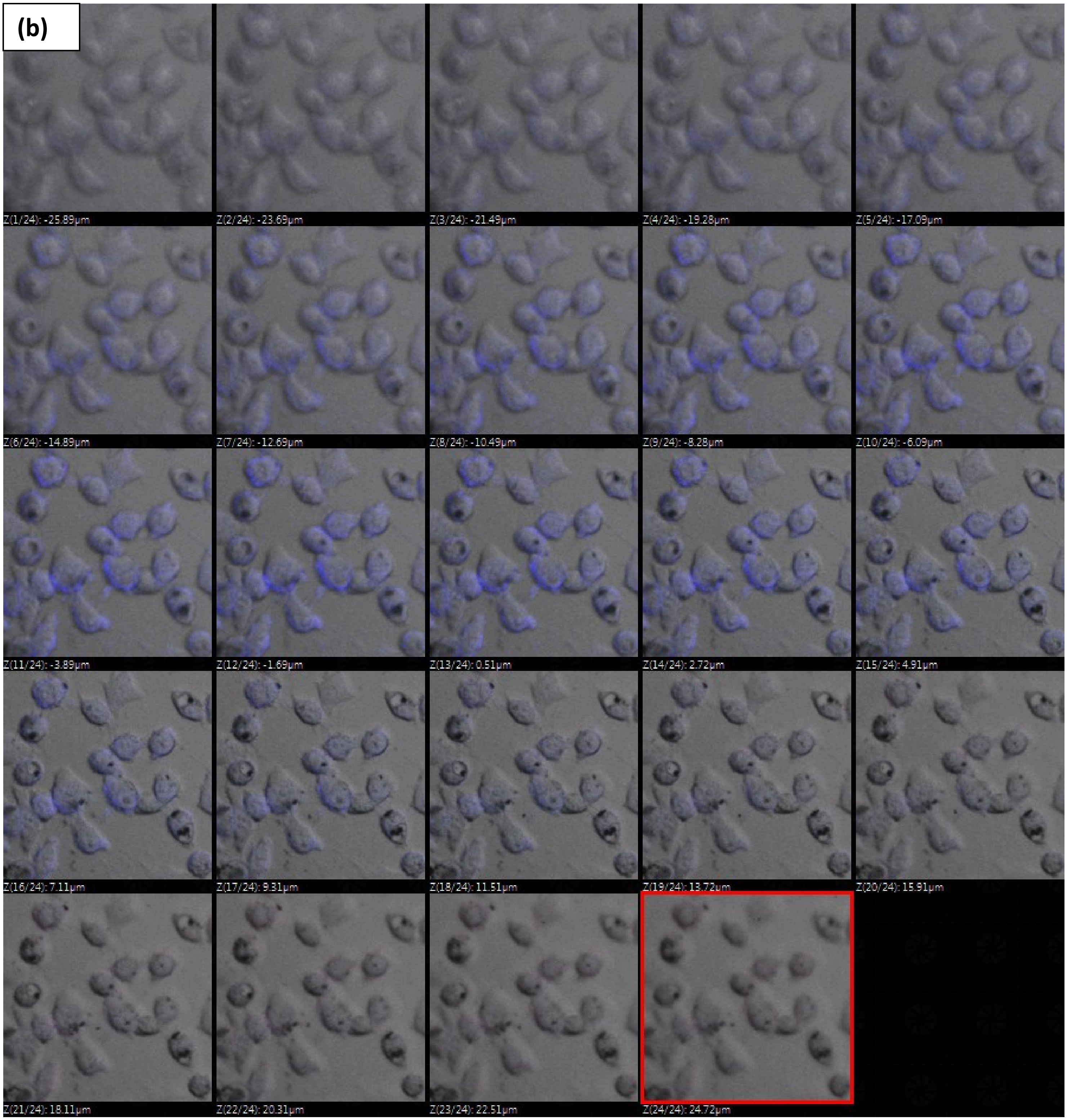
(b)
Z(1/24): -25.89μm
Z(2/24): -23.69μm
Z(3/24): -21.49μm
Z(4/24): -19.28μm
Z(5/24): -17.09μm
Z(6/24): -14.89μm
Z(7/24): -12.69μm
Z(8/24): -10.49μm
Z(9/24): -8.28μm
Z(10/24): -6.09μm
Z(11/24): -3.89μm
Z(12/24): -1.69μm
Z(13/24): 0.51μm
Z(14/24): 2.72μm
Z(15/24): 4.91μm
Z(16/24): 7.11μm
Z(17/24): 9.31μm
Z(18/24): 11.51μm
Z(19/24): 13.72μm
Z(20/24): 15.91μm
Z(21/24): 18.11μm
Z(22/24): 20.31μm
Z(23/24): 22.51μm
Z(24/24): 24.72μm

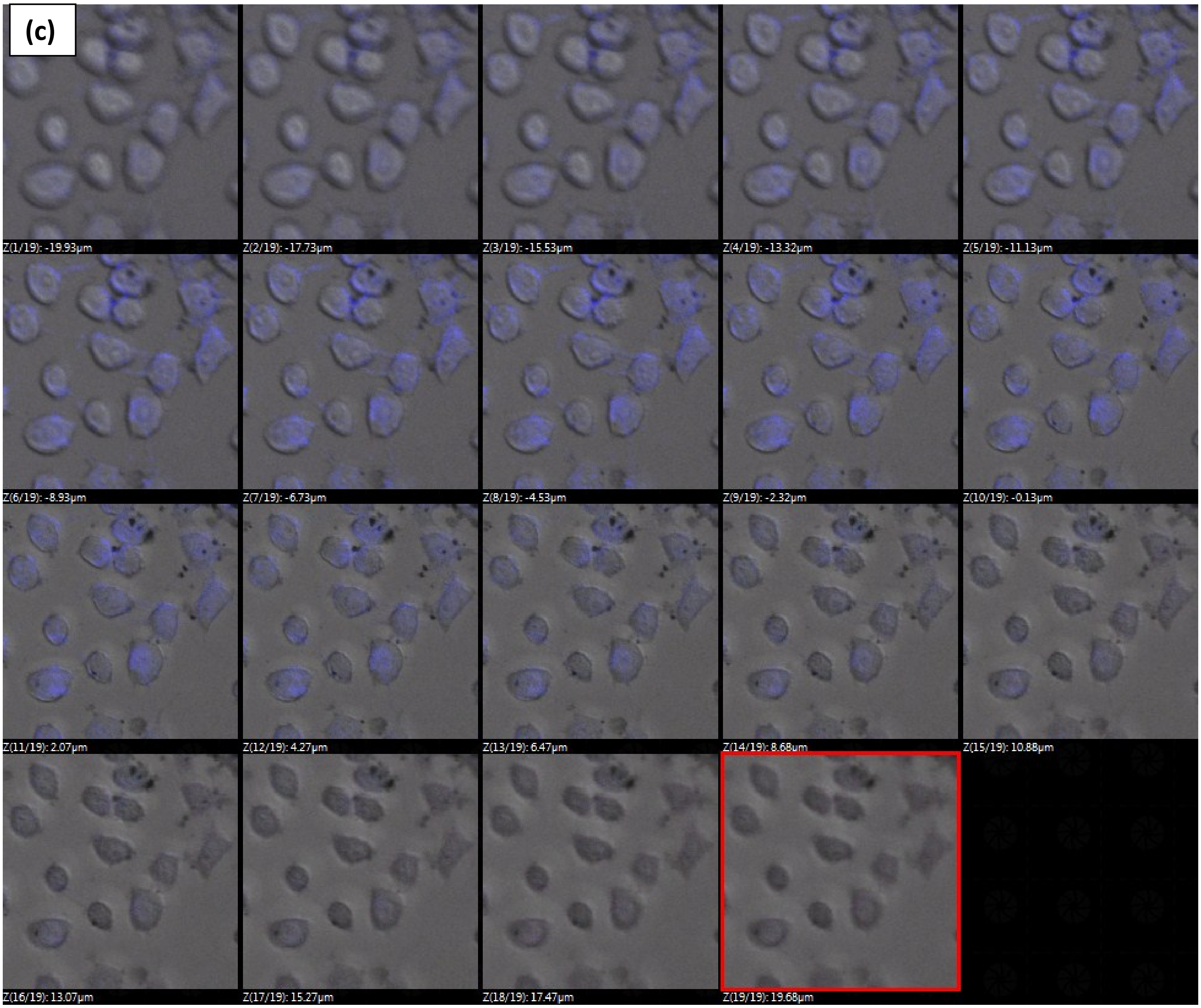

Figure S3 Real-time fluorescence imaging of cell apoptosis process in HeLa cells incubated with (a) PLU,(b)POPD, (c) POPD/PLU-20/80 for 4 h
